# Atmospheric Induction Direct Chromium Alloying of Steel Using FeAlSiCa as a Metallothermic Reductant

**DOI:** 10.3390/ma19061111

**Published:** 2026-03-12

**Authors:** Amankeldy Akhmetov, Yerbolat Makhambetov, Arnat Smagulov, Zhadiger Sadyk, Ruslan Toleukadyr, Sailaubai Baisanov

**Affiliations:** 1 Zh. Abishev Chemical-Metallurgical Institute, Ermekov Street 63, Karaganda 100009, Kazakhstan; arnatsmagulov@gmail.com (A.S.); sadzhad03@gmail.com (Z.S.);; 2 QazMetals Engineering, Mukanov Street 61/2, 198, Karaganda 100024, Kazakhstan; ruslan-94kz@mail.ru

**Keywords:** steel, stainless steel, corrosion-resistant steel, chromium, ferrochrome, alloying, induction furnace, metallurgy

## Abstract

**Highlights:**

- Direct Cr alloying in an induction furnace using oxide raw materials is demonstrated.
- Cr recovery up to 83% is achieved under atmospheric induction melting.
- DRI content strongly influences Cr recovery and element distribution in steel.
- Direct alloying avoids ferrochrome production and reduces overall chromium losses.
- The process works without vacuum or gas protection, simplifying industrial adoption.
- The method is suitable for small and medium steelmaking operations.

**Abstract:**

The study investigates the technology of direct Cr alloying of steel in an induction furnace using Cr-containing oxide raw materials and an FeAlSiCa metallothermic reducing agent under atmospheric conditions. The experimental design included four charge variants: scrap-based, DRI-based, A-series (50% scrap/50% DRI), and B-series (75% scrap/25% DRI). For A-series and B-series, the FeAlSiCa content was varied from the baseline value to reduced levels of −10% and −20%. The results demonstrate that Cr recovery strongly depends on the metallic component of the charge. The highest Cr recovery (up to 83%) was consistently achieved for the DRI-based charge, while mixed charges showed intermediate values depending on the DRI fraction and reducer amount. Reduction in FeAlSiCa content led to a decrease in Si transfer to steel, but was accompanied by lower Cr recovery. The produced steels were characterized by a uniform distribution of alloying elements, low impurity levels (S, P < 0.03%), and the formation of a dense, non-disintegrating slag. The results confirm that direct Cr alloying in an induction furnace can be effectively implemented under atmospheric conditions without vacuum or protective gas atmosphere, while the presence of DRI plays a key role in enhancing Cr assimilation.

## 1. Introduction

A large number of methods for producing alloyed steels can be classified according to the main technological unit used during steel melting. For example, there are technologies for producing alloyed steels based on converters, open-hearth furnaces, electric arc furnaces, and induction furnaces, as well as combined processing routes involving secondary metallurgy units, etc. [1,2,3]. Despite the diversity of approaches, a common feature is the introduction of alloying elements predominantly in metallic form, most often as iron-based alloys such as FeSi, FeMn, FeV, FeCr, and others [4]. The use of ferroalloys is widespread due to their ease of transportation, good processability during addition, and stable assimilation of alloying elements [4]. However, the application of ferroalloys leads to a number of problems, including the presence of undesirable impurities and the high cost of producing high-quality grades (for example, low-carbon (LC) ferroalloys) [4,5].

Despite the development of new corrosion protection methods [6,7,8,9,10], stainless steel (corrosion-resistant steel) remains the main corrosion-resistant structural material in mechanical engineering.

For corrosion-resistant steel grades, chromium is the key alloying element. It is well known that when the Cr content in an alloy exceeds approximately 12%, a stable passive oxide film forms, providing high corrosion resistance; steels with such Cr levels are classified as stainless steels. A widely used technological route for producing corrosion-resistant steel is based on the use of FeCr. This ferroalloy is added to the charge of an electric arc furnace, most often together with steel scrap, and the high-carbon steel obtained after melting is subsequently refined, primarily by decarburization, in secondary metallurgy units, for example in an AOD (argon–oxygen decarburization) converter [11,12]. Such a technological route involves double melting of raw materials (first, ore during FeCr production, and then ferrochrome itself during steelmaking) and additional operations for C removal, which increases energy and resource consumption [13]. FeCr production itself is extremely energy intensive: approximately 3–4.7 MWh of electrical energy is required to produce one ton of this alloy, since the process is based on high-temperature reduction of chromite ore in a submerged arc furnace [14]. In addition, Cr losses due to incomplete reduction can reach up to 30% [15]. Subsequent oxygen blowing of the melt to reduce C content leads to additional oxidation of Cr and its transfer to the slag phase, and part of this Cr can be returned to the metal only through the addition of deoxidizers, such as expensive FeSi [16,17]. Moreover, Cr-containing slags pose an environmental hazard due to the presence of hexavalent Cr compounds [18].

Thus, the conventional route for producing corrosion-resistant steel using ferrochrome is characterized by high energy consumption, significant losses of the alloying element, and the formation of harmful by-products.

In view of these issues, alternative methods for Cr alloying of steel are being developed with the aim of eliminating the separate stage of ferroalloy production. One of the promising approaches is the technology of direct alloying, in which Cr compounds are introduced directly into the steel melt not in the form of a finished metallic alloy, but in a non-metallic form (for example, as oxides), and are then reduced in situ to the metallic state. It can be assumed that direct alloying will reduce ferroalloy consumption, decrease energy input, and increase the yield of the target element by minimizing oxidation losses, as well as reduce the volume of harmful waste.

In the case of chromium, the direct alloying technology involves adding Cr-containing raw materials (for example, chromite concentrate) to the melt together with a suitable reducing agent, resulting in in situ reduction of Cr and its dissolution in steel. Various versions of this process have been implemented under laboratory and pilot-scale conditions. For example, it has been shown that the use of a charge consisting of chromite ore, metallic Fe, and C as a reducing agent ensures a high degree of Cr transfer into steel, on the order of 90%, provided that the slag composition is properly adjusted and a sufficiently high reducing potential of the environment is maintained [19]. It has also been established that briquetting oxide raw materials together with Fe-containing components and a carbonaceous reducing agent improves the completeness of the reaction [20]. When briquettes composed of a mixture of chromite concentrate, mill scale, and coke are added to the steel charge in an induction furnace, steel can be alloyed with Cr with virtually no losses; the degree of Cr recovery into the metal reaches approximately 93–100% under optimal melting conditions [20]. A vacuum–carbon process is also considered a promising reduction method [21]. Recent studies have shown that when Cr_2_O_3_ is introduced into the melt and reduced by powdered C under reduced pressure, a Cr yield exceeding 95% can be achieved, reaching approximately 96–97% under optimal parameters, while simultaneously reducing the oxygen content and the amount of non-metallic inclusions in steel [21]. The silicothermic process has also made it possible to achieve a Cr recovery rate of up to 95.4% during direct alloying of steel in an induction furnace [22].

These results indicate that direct Cr alloying can successfully replace or supplement the traditional use of FeCr, reducing costs and environmental risks.

Special attention in the development of this technology is given to induction furnaces as a basis for direct alloying. Induction melting units are widely used for producing alloyed and stainless steels, especially in foundries and small-scale industrial facilities. Induction melting is characterized by a high purity, as there is no contact between the melt and fuel combustion products, which reduces metal oxidation. However, induction furnaces do not provide opportunities for active melt refining, particularly for the removal of excess C by blowing [23]. Therefore, when producing corrosion-resistant steel grades in induction furnaces, carefully selected charges with minimal impurity and C contents are used [24,25].

Against the background of these limitations, the search for a technologically and economically efficient method of direct Cr alloying specifically under induction melting conditions becomes especially relevant. In the present study, it is proposed to use an FeAlSiCa alloy as a reducing agent, which is obtained mainly from technogenic waste [14]. This alloy exhibits pronounced deoxidizing and reducing properties and is capable of providing an exothermic heating effect, while remaining accessible and more affordable compared to other metallic reducing agents [14]. It is fundamentally important that the process is carried out under atmospheric conditions, without the use of vacuum, which simplifies its industrial implementation.

This approach has the potential to produce corrosion-resistant alloys of the required composition without a separate energy-intensive stage of FeCr production, thereby improving both the economic efficiency and environmental performance of the process. Accordingly, the present study is aimed at examining the technology of direct Cr alloying of steel in induction furnaces, moving from a general overview of methods toward the formulation of tasks for its practical implementation.

## 2. Materials and Methods

The starting materials used in this study can be conditionally divided into two groups. The first group consisted of steel scrap and direct reduced iron (DRI) sponge, obtained by hydrogen reduction in rolling mill scale. The second group included Cr-concentrate, the FeAlSiCa reducing agent, and flux in the form of lime. The selection of charge components for the second group was based on previously conducted studies on the production of LCFeCr in an induction furnace [26].

The chemical composition of all listed materials is presented in Table 1 and Table 2.

To determine the chemical composition of the materials listed in Table 1 and Table 2 and of the obtained steel samples, all samples were ground to a particle size of <75 µm and dissolved by acid digestion using mixtures of HCl, HNO3, HF, and other acids where required. Chemical reagents for analytical chemistry methods were supplied by IP Mendeleev (Karaganda, Kazakhstan). Some elements were quantified using gravimetric and volumetric titration techniques according to standard procedures. Calcination losses were determined by heating the samples at 1000 °C for 1 h. Primarily, various GOST standards were applied [27,28,29].

During subsequent melting experiments, DRI, Cr-concentrate, FeAlSiCa, and lime had particle sizes of <120 µm, while the steel scrap was used in the form of rod cuttings with a diameter of approximately 1 cm and a height of 0.5–1.5 cm.

Based on the above-mentioned previous studies [26], the composition of the initial charge was calculated. The main principles of the calculation were the complete transfer of the main charge components into the steel composition, and the stoichiometric quantities of reducing agents were determined based on the total oxygen content in the oxide components of the Cr-concentrate. The resulting charge compositions are presented in Table 3.

Previous studies have shown that a 10% excess of FeAlSiCa is an optimal condition [26], which was therefore selected as the baseline for the Scrap-based, DRI-based, A1, and B1 charges. Subsequently, the amount of the reducing agent was decreased by 10% for the A2 and B2 charges and by 20% for the A3 and B3 charges. Lime was added at a ratio of 1.8 CaO per 1 SiO_2_ formed during smelting, in order to bind Si, P, and S impurities [26].

Smelting was carried out in an induction furnace with a transformer power rating of 16 kVA under atmospheric air conditions.

Prior to melting, the charge components (steel scrap or DRI, Cr-concentrate, FeAlSiCa, and lime) were weighed according to the compositions listed in Table 3. The powdered components were used with a particle size of <120 µm, while the metallic scrap was introduced as rod cuttings.

The charge was loaded into a ceramic crucible (99.9% Al_2_O_3_) in the following sequence: first, the metallic component (scrap and/or DRI) was introduced, followed by the premixed oxide and reducer mixture (Cr-concentrate, FeAlSiCa, and lime), after which the remaining metallic portion was added on top.

The ceramic crucible was placed inside a graphite crucible, which was inductively heated and served as the primary heat-transfer medium to the charge.

Heating was performed by gradually increasing the electric current up to 600 A. Upon reaching the maximum current, the melt was held for 6 min.

Temperature was measured using a W–Re thermocouple housed in an alumina sheath. The thermocouple tip was positioned at the center of the crucible. The thermocouple was calibrated according to the manufacturer’s specifications prior to experiments.

After completion of the holding period, the system was allowed to cool in air within the crucible. Upon complete cooling, the crucible was mechanically destroyed, and the metal ingot and slag were separated. Metallic droplets recovered during post-mortem separation were included in the metal yield evaluation.

The resulting metal samples were examined using a Zeptools ZEM20 (ZEPTOOLS, Tongling City, China) scanning electron microscope (SEM) equipped with an Oxford energy-dispersive spectroscopy (EDS) detector (Oxford Instruments, Abingdon, UK). EDS maps were acquired at 15 kV and 3 nA; the dwell time per pixel and number of frames were kept constant for all samples. Selected elements (S, P, N, and C) and slag samples were analyzed using standard analytical chemistry techniques, including the aforementioned acid digestion, gravimetric, and volumetric titration methods [30,31,32,33].

The recovery of Cr and Fe was calculated using Equation (1):

(1) $$R_{i}\left(\%\right)=\frac{m_{metal}w_{i,\;meetal}+m_{drops}w_{i,\;drops}}{\Sigma_{k}m_{k}w_{i,\;k}}\times 100$$

where

i = Fe and Cr

m_metal_—is the mass of the main metal ingot;

m_drops_—is the mass of metallic droplets;

w—is the mass fraction of the element in the corresponding charge component k.

## 3. Results

### 3.1. Steel Smelting from Scrap-Based and DRI-Based Charges

**Figure 1:**
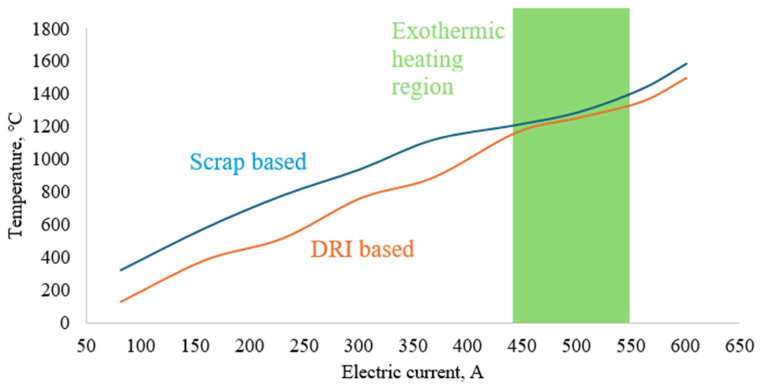
Temperature profile of induction melting using scrap-based and DRI-based charges.

The use of steel scrap for steel production in an induction furnace is a well-known approach and is also applied in the technology considered in this study. Steel scrap (or DRI) and the charge mixture for Cr smelting were loaded into the crucible. The crucible was then placed into the working zone of the induction furnace. Heating of the charge was carried out by gradually increasing the electric current up to 600 A, while recording the temperature inside the crucible. A W–Re thermocouple housed in an alumina sheath was used. The thermocouple tip was placed at the center of the crucible. The thermocouple was calibrated according to supplier specifications; uncertainty is within the typical range for W–Re thermocouples at the operating temperatures. The results are presented in Figure 1. The temperature profiles shown represent average values obtained from three experiments (*n* = 3), with a relative deviation of approximately 3–5%.

Figure 1 demonstrates a more efficient heating of the scrap-based charge. Up to a current of 450 A, the temperature difference reaches approximately 200–250 °C. At currents of 450 A and above, this difference decreases significantly to 50–100 °C. This behavior is associated with the fact that, at a current of about 450 A, temperature fluctuations accompanied by short-term heating up to ~1400 °C are observed in both charge variants due to exothermic heat release during oxidation of FeAlSiCa. Previously, during induction smelting of LCFeCr under similar reduction conditions using FeAlSiCa, temperatures as high as ~2100–2200 °C were reported [26]. In the present case, the difference is attributed to a lower content of the reducing agent and to the more localized nature of such overheating, with the generated heat being distributed within a colder melt whose base consists of scrap and DRI. At this stage, a liquid phase is formed.

Due to the closed crucible configuration, the onset of slag formation could not be visually observed in real time; slag formation was assessed post-mortem after cooling and crucible breakage.

When the maximum current of 600 A is reached and an isothermal holding time of 6 min is applied, the maximum temperatures are 1585 and 1497 °C for the scrap-based and DRI-based charges, respectively.

**Figure 2:**
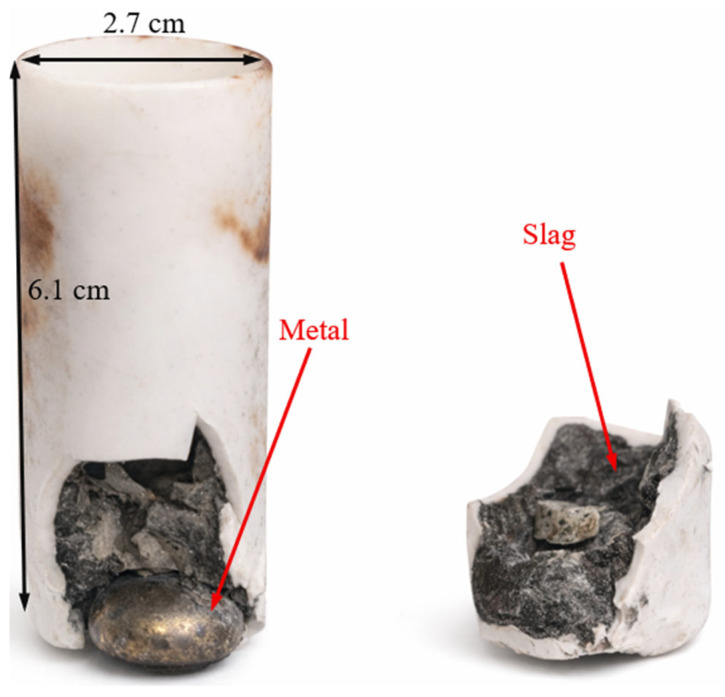
Typical appearance of induction melting products.

Figure 2 shows the typical appearance of the smelting products in a fractured crucible after melting.

From Figure 2, the formation of a solid metal ingot with a relatively low amount of metallic droplets (no more than 1.1–2.1% of the total metal mass) can be observed. The slag is dense and stone-like and does not undergo self-disintegration even after more than 30 days of observation. Similar results were obtained for all charge variants.

**Figure 3:**
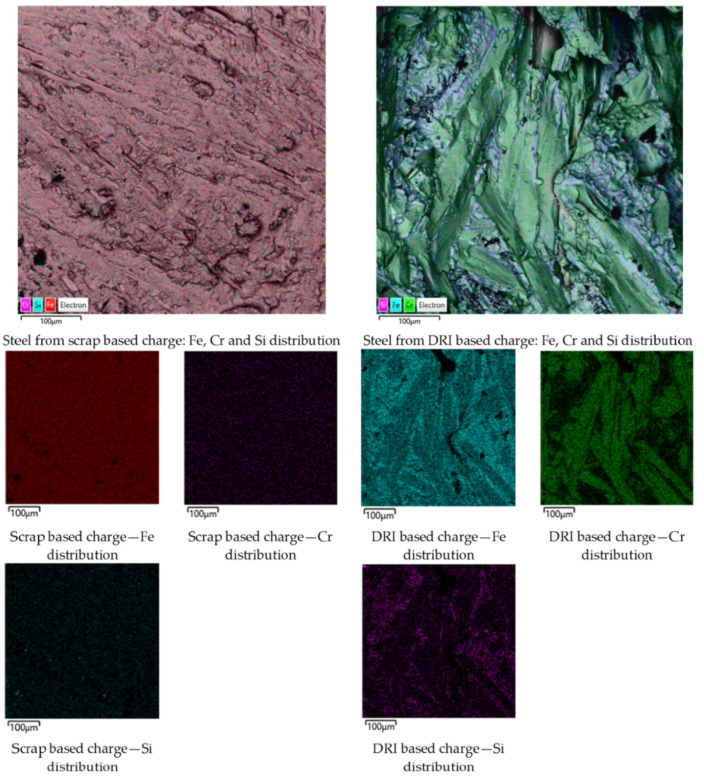
Elemental distribution maps of Fe, Cr, and Si on the fracture surfaces of steels produced from scrap-based and DRI-based charges.

Fracture surfaces of the samples were examined by SEM coupled with EDS. Typical results in the form of elemental distribution maps are shown in Figure 3.

From Figure 3, a uniform distribution of alloying elements over the fracture surface can be observed for the scrap-based charge. In contrast, for the DRI-based charge, pronounced segregations of Cr and Fe–Si-rich regions are evident. The chemical compositions of steels obtained from the scrap-based and DRI-based charges are presented in Table 4.

Similar results were consistently obtained in repeated melts. Taking into account the mass and composition of the initial charge, the mass of the obtained metal, and its chemical composition, the Fe recovery from scrap/DRI amounted to 95% and 80%, respectively. The Cr recovery was 59% for the scrap-based charge and 83% for the DRI-based charge.

Such differences in chromium recovery can be attributed to two main factors. The first is the loss of exothermic heat required for Cr_2_O_3_ reduction. It is likely that colder steel scrap absorbs the released energy more efficiently than DRI due to its higher density and, consequently, higher thermal conductivity. In contrast, DRI powder particles distribute the released reaction energy more uniformly throughout the mixture volume. The second and more significant factor is the beneficial effect of DRI powder, which promotes in situ dissolution of newly formed Cr into the Fe phase and the formation of Fe–Cr solid solutions and mixed carbide phases. Although the amount of these carbides is small, their presence is expected due to the impurity C contained in FeAlSiCa and steel scrap. This behavior reduces the activity of Cr in the system and thermodynamically facilitates its further reduction [19].

This interpretation is consistent with the observed elemental distribution maps.

Despite exhibiting lower Cr recovery, the scrap-based samples show a more uniform distribution of alloying elements. This can be explained by the fact that dense metallic scrap facilitates rapid dissolution of the reduced Cr into the liquid metal phase, even when the overall reduction efficiency is limited by heat absorption.

In contrast, although DRI promotes more efficient Cr reduction, the finely dispersed reaction environment may lead to localized enrichment of Cr before complete homogenization in the melt, resulting in the observed segregated distribution patterns.

Based on these results, it was decided to investigate the possibility of direct steel alloying using different combinations of steel scrap and DRI and varying amounts of the reducing agent, in order to prevent excessive Si transfer into the metal. This is particularly relevant because the exclusive use of DRI may be economically disadvantageous due to its higher cost.

### 3.2. Steel Smelting from A1, A2, and A3 Charges

A distinctive feature of the A-series charges is the replacement of 50% of steel scrap with DRI while introducing the optimal amount of FeAlSiCa (A1), as well as charges with a 10% (A2) and 20% (A3) deficiency of the reducing agent.

**Figure 4:**
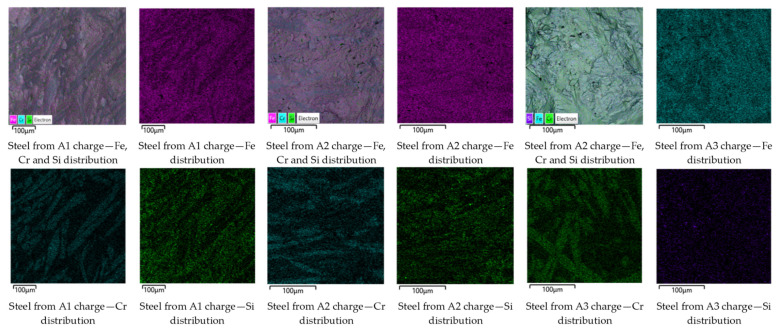
Elemental distribution maps of Fe, Cr, and Si on fracture surfaces of steels produced from A1–A3 charges (50% scrap / 50% DRI) with decreasing FeAlSiCa content (baseline, −10%, and −20%). The overall metal yield for the A1, A2, and A3 charge variants was 86%, 85%, and 87%, respectively, with an average relative error of 2–4% in repeated experiments. This indicates practically identical overall metal recovery in all cases.

Figure 4 shows typical elemental distribution maps on the fracture surfaces of representative steel samples. From these maps, a distribution pattern very similar to that observed for steel produced from the DRI-based charge can be seen. Cr is predominantly concentrated in localized regions, whereas Fe and Si form the alloy matrix. A decrease in FeAlSiCa content leads to a gradual reduction in Si concentration. For the A3 charge, a significantly lower Si content is observed, manifested by a more uniform and dispersed distribution over the entire fracture surface.

The chemical composition was determined by EDS for Fe, Cr, and Si, and by classical analytical chemistry methods for C, S, and P. The summarized results for typical samples shown in Figure 4 are presented in Table 5.

Considering the compositions presented in Table 4, it becomes evident that the Fe recovery from the main charge components in the form of scrap/DRI is lower. Recalculation based on the actual mass of the obtained metal shows that Fe recovery averaged 78–82%, regardless of the reducing agent content, including data from repeated experiments. The average Cr recovery was 75%, 65%, and 62% for the A1, A2, and A3 charges, respectively. Repeated experiments showed a relative error of 5–6%.

### 3.3. Steel Smelting from B1, B2, and B3 Charges

A distinctive feature of the B-series charges is the replacement of 25% of steel scrap with DRI while introducing the optimal amount of FeAlSiCa (B1), as well as charges with a 10% (B2) and 20% (B3) deficiency of the reducing agent.

**Figure 5:**
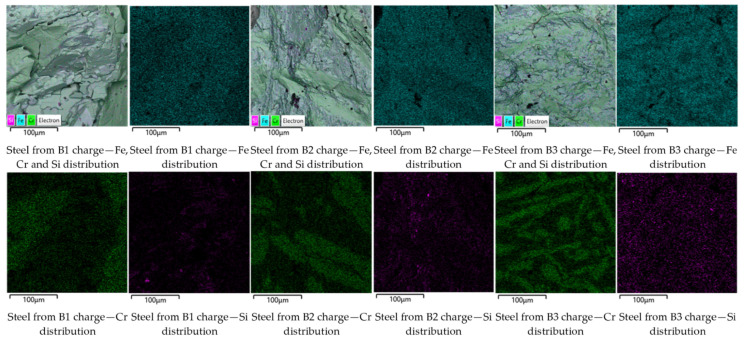
Elemental distribution maps of Fe, Cr, and Si on fracture surfaces of steels produced from B1–B3 charges (75% scrap / 25% DRI) with decreasing FeAlSiCa content (baseline, −10%, and −20%).

Figure 5 presents typical elemental distribution maps on the fracture surfaces of the obtained steel samples. From these maps, a similar characteristic distribution of chromium and a clear trend in silicon distribution depending on the FeAlSiCa content can be observed.

Table 6 presents the chemical analysis data for steels produced from the B1–B3 charges, determined by EDS for Fe, Cr, and Si and by analytical chemistry methods for C, S, P and N (N was not detected in the metal and therefore is not included in the table).

Calculations analogous to those performed for the A1–A3 charges were carried out, taking into account the mass of metal in the charge in the form of scrap, DRI, and Cr-concentrate, as well as the mass and composition of the metal obtained after smelting. According to these calculations, Fe recovery ranged from 85% to 95%. The average Cr recovery was 65%, 63%, and 60% for the B1, B2, and B3 charges, respectively. Repeated experiments showed a similar relative error of 6–7%.

## 4. Discussion

The experimental results confirm the feasibility of direct Cr alloying of steel in an induction furnace using Cr-containing oxide raw material and the FeAlSiCa reducing agent. The process of direct Cr alloying in an induction furnace, employing a charge that previously enabled the production of LCFeCr under nearly identical conditions, is characterized by a certain degree of complexity. In practice, the following parameters were varied: the scrap/DRI ratio and the amount of the reducing agent. However, the obtained results for Cr recovery, in particular, demonstrate some instability, indicating the influence of additional factors.

**Figure 6:**
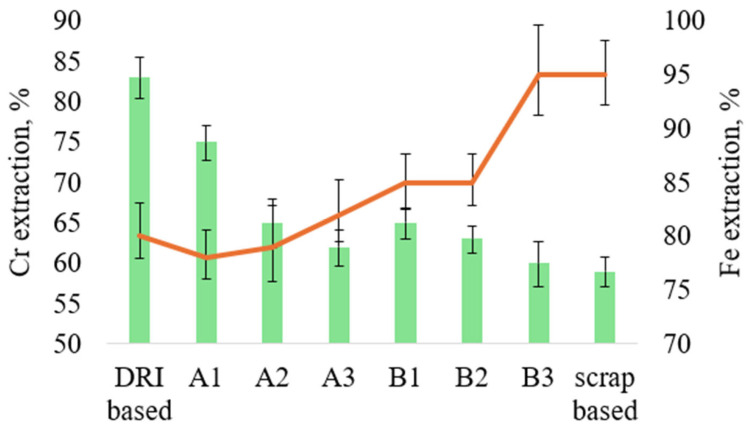
Cr and Fe extraction efficiencies for different charge variants. Green bars represent chromium (Cr) extraction, while the orange line indicates iron (Fe) extraction.

The proportion of DRI (iron powder) has a significant effect on Cr recovery. Figure 6 presents the Cr and Fe recovery values for the investigated charge variants.

The Cr recovery values are comparable to those achieved during standalone LCFeCr production. In the standalone process, Cr recovery into steel from Cr-concentrate was about 65% when using an FeAlSiCa content that prevented excessive Si transfer into the metal [26]. A decrease in the FeAlSiCa amount leads to a reduction in Si content in the metal, which was also previously observed during standalone LCFeCr production in an induction furnace. For comparison, during FeCr production in closed submerged arc furnaces (SAF), the upper reported range of Cr recovery is 83–88% when using pre-oxidized pellets, whereas for conventional SAF smelting of unscreened chromite this value is typically 70–75% [15,34].

High Cr recovery values of up to 90% for a mixture of chromite ore and Fe scrap under carbothermic reduction conditions in an induction furnace were reported by Hu et al. [19]. It was also noted that Fe powder is more favorable in terms of Cr recovery. At the same time, in that study [19], smelting was carried out under an Ar atmosphere. Similarly, higher Cr recovery during direct alloying via the silicothermic process, reaching up to 95.4% [22], was achieved during induction melting in an Ar atmosphere with a purity of 99.999%.

These findings indicate a strong influence of the oxidizing nature of the surrounding atmosphere. This effect is further compounded by the oxidized nature of the initial raw materials and the high susceptibility of FeAlSiCa components to oxidation. During the production of corrosion-resistant steel in an electric arc furnace (EAF), slag containing up to 2–10% Cr_2_O_3_ is formed [16]. Under vacuum conditions, in a carbothermic process, Cr recovery can indeed exceed 95% due to a reduction in the partial pressure of CO [21]. However, the use of vacuum significantly complicates the technology and increases its cost.

The maximum Cr recovery of approximately 83% obtained using the DRI-based charge is associated with the limitations of the experimental smelting conditions described above. Slag basicity is also an important factor, as higher Cr recovery is known to be favored by higher slag basicity. The calculated average chemical composition of slag samples obtained from different charge variants is presented in Table 7.

In the present study, the slag basicity for all charge variants was in the range of 0.83–0.97, which is consistent with the values reported in baseline studies on LCFeCr production in an induction furnace using FeAlSiCa [26]. For LCFeCr, slag basicity values typically fall within the range of 0.8–1.4 [14], indicating the potential for further increasing slag basicity in future studies to improve Cr recovery.

Although higher slag basicity is generally reported to favor Cr recovery, the present results indicate that within the investigated basicity range (0.83–0.97), no clear linear relationship between basicity and Cr recovery can be observed. For example, the highest Cr recovery (83%) was obtained for the DRI-based charge with a basicity of 0.86, whereas charges with higher basicity values (e.g., B1, B = 0.97) did not demonstrate proportionally higher Cr recovery.

This suggests that under atmospheric induction melting conditions, the metallic charge composition and thermal factors exert a stronger influence on Cr recovery than slag basicity alone.

It should also be noted that when FeCr is added as an alloying agent, significant Cr losses are unavoidable. Up to 30% of Cr is lost to slag during alloy production [15], with additional losses occurring during subsequent steelmaking [16]. Direct alloying eliminates this double oxidation of chromium. In the present experiments, the overall chromium recovery into the metal reached approximately 80%. Thus, the total chromium yield over the entire technological chain increases, while metallurgical losses are reduced.

For all charge variants, a low content of detrimental impurities in the obtained steel samples was observed (S, P < 0.03%), indicating the effectiveness of the refining reactions involved, for example those associated with lime addition to bind S and P into the slag. During FeCr smelting under strongly reducing conditions (low oxygen activity), a thermodynamically favorable environment is created for N dissolution and the formation of stable Cr nitrides [26]. In contrast, during direct alloying of steel, the Cr content is significantly lower, and the presence of O_2_ and slag limits the kinetics of N absorption; moreover, any N that does enter the melt is diluted within the metal bath and is not fixed as nitride phases, remaining below the detection limit.

In addition to enabling direct Cr alloying of steel, which opens prospects for the direct production of corrosion-resistant steels, an important advantage of the proposed approach is slag stability. It is well known that FeCr slags can contain dicalcium silicate, which undergoes self-disintegration upon cooling due to the γ-Ca_2_SiO_4_ phase transformation [35]. Based on the stable condition of the slags during storage for more than 30 days, this effect was successfully avoided in the present study. Similar results were previously observed during standalone LCFeCr production in an induction furnace using FeAlSiCa [26].

Unlike many previously reported direct alloying studies conducted under Ar or vacuum conditions [19,21,22], the present experiments were performed under atmospheric air. The presence of O_2_ in the surrounding atmosphere inevitably increases the risk of partial oxidation of the reducing agent and Cr, which may limit the maximum achievable recovery. This represents a thermodynamic and kinetic penalty compared to inert or vacuum conditions.

However, the elimination of protective gas or vacuum systems significantly simplifies the process and reduces capital and operational costs. From an industrial perspective, especially for small and medium-scale induction furnaces, operation under atmospheric conditions may offer a favorable trade-off between slightly lower Cr recovery and substantially improved technological simplicity and economic feasibility.

## 5. Conclusions

The present study demonstrated the feasibility of atmospheric direct Cr alloying of steel in an induction furnace using Cr-containing oxide raw material and FeAlSiCa as a metallothermic reductant. Based on the experimental results, the following conclusions can be drawn:

- The best-performing charge was the DRI-based composition, which achieved a maximum chromium recovery of 83% under atmospheric induction melting conditions (16 kVA furnace, 600 A current, 6 min holding time, Al_2_O_3_ crucible placed inside a graphite susceptor).
- Scrap-based charges resulted in lower Cr recovery (59%) but exhibited more uniform elemental distribution due to enhanced dissolution of reduced chromium in the liquid metal phase.
- In mixed charges (A- and B-series), Cr recovery ranged between 60–75% depending on the DRI fraction and FeAlSiCa content, confirming the dominant role of metallic charge composition over slag basicity within the investigated range (0.83–0.97).
- Decreasing the FeAlSiCa content reduced i transfer to the metal (down to 0.06% in A3) but was accompanied by a reduction in Cr recovery, indicating a trade-off between reduction efficiency and Si control.
- All obtained steels were characterized by low impurity levels (S, P < 0.03%) and the formation of dense, non-disintegrating slag.

Although Cr recovery under atmospheric conditions is moderately lower than values reported for argon or vacuum-assisted processes, the elimination of protective gas or vacuum systems significantly simplifies the process and reduces equipment complexity.

Further improvement of Cr recovery and melt homogeneity may be achieved through:

- Optimization of slag basicity beyond the investigated range (B > 1.0) to enhance Cr retention in the metallic phase;
- Extension of holding time to promote complete dissolution and homogenization of reduced Cr;
- Application of cover gases or controlled O_2_ potential to reduce oxidative losses under atmospheric operation;
- Implementation of staged or controlled feeding of oxide and reductant components to improve reaction control and temperature distribution.

## Figures and Tables

**Table 1:** Chemical composition of the steel scrap and DRI, wt.%.

	Fe	Cr	Si	Al	Ca	C	S	P	Mn	Ti	V
Scrap	bal.	0.15	0.20	-	-	0.20	0.05	0.04	0.55	-	-
DRI	bal.	0.10	0.12	-	-	0.05	0.03	0.02	0.12	-	-
FeAlSiCa	bal.	-	67.05	13.38	10.03	0.65	0.019	0.039	-	1.05	0.38

**Table 2:** Chemical composition of Cr-concentrate and lime, wt.%.

	Fe_2_O_3_	Cr_2_O_3_	SiO_2_	Al_2_O_3_	CaO	MgO	S	P	CL
Cr-concentrate	13.01	bal.	4.85	7.56	0.55	17.9	0.022	0.004	3.65
Lime	-	-	≤1.8	-	≥92.00	-	≤0.03	≤0.02	≤6

**Table 3:** Composition of charge variants, wt.%.

Charge	Scrap	DRI	Cr-Concentrate	FeAlSiCa	Lime
Scrap-based	35.40	-	12.50	26.70	25.40
DRI-based	-	35.40	12.50	26.70	25.40
A1	17.70	17.70	12.50	26.70	25.40
A2	18.68	18.68	13.19	25.36	24.09
A3	19.77	19.77	13.96	23.85	22.66
B1	26.55	8.85	12.50	26.70	25.40
B2	28.02	9.34	13.19	25.36	24.09
B3	29.65	9.88	13.96	23.85	22.66

**Table 4:** Chemical composition of steel samples produced from scrap-based and DRI-based charges.

Charge	Fe	Cr	Si	Ti	V	C	S	P
Scrap-based	oст.	12.38	0.65	-	-	0.20	0.021	0.030
DRI-based	oст.	24.33	1.12	0.22	0.58	0.18	0.020	0.022

**Table 5:** Chemical composition of steel samples produced from A1–A3 charges.

	Fe	Cr	Si	Ti	V	C	S	P
A1	bal.	13.89	2.49	0.14	0.35	0.15	0.020	0.031
A2	bal.	10.00	1.97	0.11	0.20	0.20	0.016	0.030
A3	bal.	16.27	0.17	0.06	0.32	0.19	0.020	0.030

**Table 6:** Chemical composition of steel samples produced from B1–B3 charges.

	Fe	Cr	Si	Ti	V	C	S	P
B1	bal.	13.97	1.99	0.17	0.34	0.18	0.020	0.030
B2	bal.	14.48	1.07	0.25	0.34	0.20	0.019	0.025
B3	bal.	16.45	0.37	0.10	0.23	0.12	0.021	0.022

**Table 7:** Calculated average chemical composition of slags.

Charge	SiO_2_	Al_2_O_3_	CaO	MgO	Cr_2_O_3_	Fe_2_O_3_	Basicity CaO + MgO/ SiO_2_ + Al_2_O_3_
Scrap-based	38.9	9.65	34.09	8.94	6.33	2.04	0.89
DRI-based	40.41	9.96	35.19	7.95	1.44	5.11	0.86
A1	38.36	10.22	36.11	8.6	2.18	4.59	0.92
A2	38.03	10.05	35	9.2	3.29	4.35	0.92
A3	40.56	9.63	32.99	8.9	3.78	3.95	0.83
B1	38.02	10.01	35.36	11	2.99	2.65	0.97
B2	41.2	9.82	34.18	9.8	3.39	2.27	0.86
B3	40.9	9.66	33.08	8.99	4.05	2.48	0.83

## Data Availability

The original contributions presented in this study are included in the article. Further inquiries can be directed to the corresponding authors.

## Abbreviations

The following abbreviations are used in this manuscript:

FeAlSiCa	Ferroaluminosilicalcium
FeCr	Ferrochrome
FeMn	Ferromanganese
FeV	Ferrovanadium
FeSi	Ferrosilicon
LC	Low-carbon
CL	Calcination losses
DRI	Direct reduced iron
SAF	Submerged-arc furnace
